# Negative Impacts of COVID-19 Induced Lockdown on Changes in Eating Behavior, Physical Activity, and Mental Health as Modified by Digital Healthy Diet Literacy and eHealth Literacy

**DOI:** 10.3389/fnut.2021.774328

**Published:** 2021-11-12

**Authors:** Tham T. Nguyen, Minh H. Nguyen, Thu T. M. Pham, Vinh-Tuyen T. Le, Tan T. Nguyen, Thuc C. Luong, Binh N. Do, Hung K. Dao, Huu C. Nguyen, Tung H. Ha, Linh V. Pham, Phuoc B. Nguyen, Hoai T. T. Nguyen, Thinh V. Do, Hung Q. Nguyen, Manh V. Trinh, Thuy T. Le, Anh L. Tra, Thao T. P. Nguyen, Kien T. Nguyen, Dung T. Phan, Khue M. Pham, Chyi-Huey Bai, Tuyen Van Duong

**Affiliations:** ^1^Faculty of Public Health, Hai Phong University of Medicine and Pharmacy, Haiphong, Vietnam; ^2^International Ph.D. Program in Medicine, College of Medicine, Taipei Medical University, Taipei, Taiwan; ^3^School of Public Health, College of Public Health, Taipei Medical University, Taipei, Taiwan; ^4^Department of Pharmacognosy-Traditional Pharmacy-Pharmaceutical Botanic, Can Tho University of Medicine and Pharmacy, Can Tho, Vietnam; ^5^Ph.D. Program in Clinical Drug Development of Herbal Medicine, College of Pharmacy, Taipei Medical University, Taipei, Taiwan; ^6^Department of Orthopedics, Can Tho University of Medicine and Pharmacy, Can Tho, Vietnam; ^7^Director Office, Can Tho University of Medicine and Pharmacy Hospital, Can Tho, Vietnam; ^8^Director Office, Military Hospital 103, Hanoi, Vietnam; ^9^Department of Cardiology, Cardiovascular Center, Military Hospital 103, Hanoi, Vietnam; ^10^Department of Infectious Diseases, Vietnam Military Medical University, Hanoi, Vietnam; ^11^Division of Military Science, Military Hospital 103, Hanoi, Vietnam; ^12^Director Office, Bac Ninh Obstetrics and Pediatrics Hospital, Bac Ninh, Vietnam; ^13^Director Office, E Hospital, Hanoi, Vietnam; ^14^Department of Thoracic and Cardiovascular Surgery, E Hospital, Hanoi, Vietnam; ^15^Director Office, General Hospital of Agricultural, Hanoi, Vietnam; ^16^Department of Pulmonary & Cardiovascular Diseases, Hai Phong University of Medicine and Pharmacy Hospital, Hai Phong, Vietnam; ^17^Director Office, Hai Phong University of Medicine and Pharmacy Hospital, Hai Phong, Vietnam; ^18^Director Office, Kien An Hospital, Hai Phong, Vietnam; ^19^Training and Direction of Healthcare Activity Center, Kien An Hospital, Hai Phong, Vietnam; ^20^Director Office, Bai Chay Hospital, Quang Ninh, Vietnam; ^21^Director Office, Quang Ninh Obstetrics and Pediatrics Hospital, Quang Ninh, Vietnam; ^22^Director Office, Quang Ninh General Hospital, Quang Ninh, Vietnam; ^23^Faculty of Medical Laboratory Science, Da Nang University of Medical Technology and Pharmacy, Da Nang, Vietnam; ^24^President Office, Da Nang University of Medical Technology and Pharmacy, Da Nang, Vietnam; ^25^Department of Physiotherapy and Rehabilitation, Da Nang University of Medical Technology and Pharmacy, Da Nang, Vietnam; ^26^Health Management Training Institute, University of Medicine and Pharmacy, Hue University, Hue, Vietnam; ^27^Department of Health Promotion, Faculty of Social and Behavioral Sciences, Hanoi University of Public Health, Hanoi, Vietnam; ^28^Faculty of Nursing, Hanoi University of Business and Technology, Hanoi, Vietnam; ^29^Nursing Office, Thien An Obstetrics and Gynecology Hospital, Hanoi, Vietnam; ^30^President Office, Hai Phong University of Medicine and Pharmacy, Hai Phong, Vietnam; ^31^Department of Public Health, College of Medicine, Taipei Medical University, Taipei, Taiwan; ^32^School of Nutrition and Health Sciences, Taipei Medical University, Taipei, Taiwan

**Keywords:** lockdown, mental health, digital healthy diet literacy, eHealth literacy, eating behavior, outpatient, physical activity

## Abstract

**Background:** The COVID-19-induced lockdown has been implemented in many countries, which may cause unfavorable changes in lifestyles and psychological health. People's health literacy, healthy diet, and lifestyles play important roles in mitigating the negative impacts of the pandemic. Therefore, we aimed to examine associations of COVID-19 lockdown with changes in eating behavior, physical activity, and mental health; and the modification effects by digital healthy diet literacy (DDL) and eHealth literacy (eHEALS) on the associations.

**Methods:** We conducted an observational study on 4,348 outpatients from 7th April to 31st May 2020. Data from 11 hospitals in Vietnam included demographic characteristics, DDL, eHEALS, eating behavior, physical activity, and mental health changes. Multiple logistic regression and interaction models were performed to examine associations.

**Results:** Patients under lockdown had a lower likelihood of having “unchanged or healthier” eating behavior (odds ratio, OR, 0.38; 95% confidence interval, 95%CI, 0.29 to 0.51; *p* < 0.001), “unchanged or more” physical activity (OR, 0.79; 95% CI, 0.69 to 0.90; *p* < 0.001), and “stable or better” mental health (OR, 0.77; 95% CI, 0.67 to 0.89; *p* < 0.001), as compared to those after lockdown. In interaction models, as compared to patients after lockdown and with the lowest DDL score, those under lockdown and with a one-score increment of DDL had a higher likelihood of having “unchanged or healthier” eating behavior (OR, 1.05; 95% CI, 1.02 to 1.07; *p* < 0.001), and “stable or better” mental health (OR, 1.02; 95% CI, 1.01 to 1.04; *p* < 0.001). Similarly, as compared to patients after lockdown and with the lowest eHEALS score, those under lockdown and with a one-score increment of eHEALS had a higher likelihood of having an “unchanged or more” physical activity (OR, 1.03; 95% CI, 1.01 to 1.05; *p* < 0.001).

**Conclusion:** The COVID-19 lockdown measure could negatively affect eating behavior, physical activity, and mental health among outpatients. Better DDL and eHEALS were found to mitigate the negative impacts of the lockdown, which may empower outpatients to maintain healthy lifestyles and protect mental health. However, this study holds several limitations that may undermine the certainty of reported findings.

## Introduction

New waves of COVID-19 outbreaks continuously re-emerged in many countries around the world (1, 2). Although vaccination programs have been deployed globally, the disproportionate distribution of vaccines (3, 4) and the emergence of new COVID-19 variants make the pandemic still uncontrolled (5, 6). Affected countries have been applying strict prevention measures such as lockdown, home confinement, and social distancing (7). Although these measures have effectively prevented the spread of the virus, it causes significant changes in people's lives, including working from home and lack of connection with family and friends (8, 9). In addition, lockdown or home confinement measures make people feel bored and isolated, negatively affecting their psychological health (10, 11). These adverse impacts on mental health can cause harmful lifestyle changes such as increasing unhealthy eating habits (12–15), sedentary behavior, or sleeping disorders (16). Recent literature also indicated that the isolation and COVID-19 lockdown had negative impacts on eating habits and emotional processing (17, 18). Furthermore, movement restrictions and difficulty accessing fresh food during the lockdown period could significantly affect people's eating patterns and physical activity habits (19–25). Therefore, it is urgent to assess the impacts of COVID-19 lockdown on changes in eating behavior, physical activity, and mental health and find protective factors that could mitigate such impacts.

The advent of the Internet and the advancement of smartphones and computer technology make it easier for people to access health information (26, 27). People could use and access web-based resources at any time to seek health advice, disease information, and check physician's consultation (28, 29). However, accessing health information and support through the Internet also has potential risks. With the ease of delivering health information through social networks and websites, it is difficult for people to recognize and evaluate which information is high-quality and reliable (30). Notably, the COVID-19 pandemic has caused an “infodemic” with a plethora of false and fake news about the disease (31, 32). This information could lead to worry and fear in the community, distrust in the government's epidemic containment efforts, and wrong health decisions (33–35). Therefore, improving skills to find, evaluate, and understand health information on the Internet is essential, especially during the COVID-19 lockdowns.

Digital healthy diet literacy (DDL) and eHealth literacy (eHEALS) have potential impacts in improving healthy lifestyles and general health during the pandemic. DDL is the ability to find, understand, evaluate, and apply healthy eating information from web-based sources to improve the eating behaviors” (36). Meanwhile, eHEALS is defined as the capacity to seek, understand, and appraise online health information and apply it to solve health issues (37). Recent literature showed that DDL was found to be associated with a higher likelihood of healthier eating, better mental health, and quality of life during the pandemic (36, 38, 39). Meanwhile, people with higher eHEALS were more likely to have better psychological health, engage in positive health-related behaviors (e.g., healthy eating, physical activity) (40–43). In addition, previous research also indicated that DDL and eHEALS could help to mitigate the negative impacts of COVID-19 on quality of life among outpatients (39). Therefore, DDL and eHEALS roles should be investigated and paid more attention to in the lockdown period.

During the pandemic, people seeking medical care have faced many challenges, such as limited access to medical care, delays in treatment, fear of COVID-19 infection, and worry about their health (35, 44, 45). As a result, maintaining healthy lifestyles and stabilizing mental health is essential to improve their health and overcome difficulties, especially in the lockdown period (46). Therefore, we conducted this study to examine the associations of COVID-19 induced lockdown with changes in eating behavior, physical activity, and mental health; and further determine whether DDL and eHEALS could modify these associations among outpatients from 11 hospitals across Vietnam.

## Materials and Methods

### Study Design, Settings, and Sampling

A cross-sectional survey was conducted in outpatients from eleven hospitals across Vietnam. Participants were recruited at selected hospitals using the convenience sampling method from 7th April to 31st May 2020. The Vietnamese Government announced a nationwide lockdown from 1 to 22 April to contain the spread of the COVID-19 pandemic (47, 48). During the lockdown period, all people are ordered not to leave their homes except for emergency cases, buying essential goods or medicine, and are prohibited from gathering more than two people in public. If going outside, people must wear a mask and keep a safe distance of two meters from others. The stringent social distancing and isolation measures yielded positive results, and no confirmed COVID-19 cases were recorded in Vietnam from mid-April to the end of May 2020 (49). After 22 April 2020, Vietnam began to gradually lift strict movement restrictions, including allowing businesses activities and schools in many parts of Viet Nam to re-open, and resuming domestic travel across the country. However, epidemic prevention measures continued to be implemented according to the “5K Rule” of the Vietnam Ministry of Health, including wearing a mask when going out, washing hands regularly with soap or sanitizer, keeping a safe distance from others, not gathering in crowds, and making a medical declaration (50).

**Figure 1:**
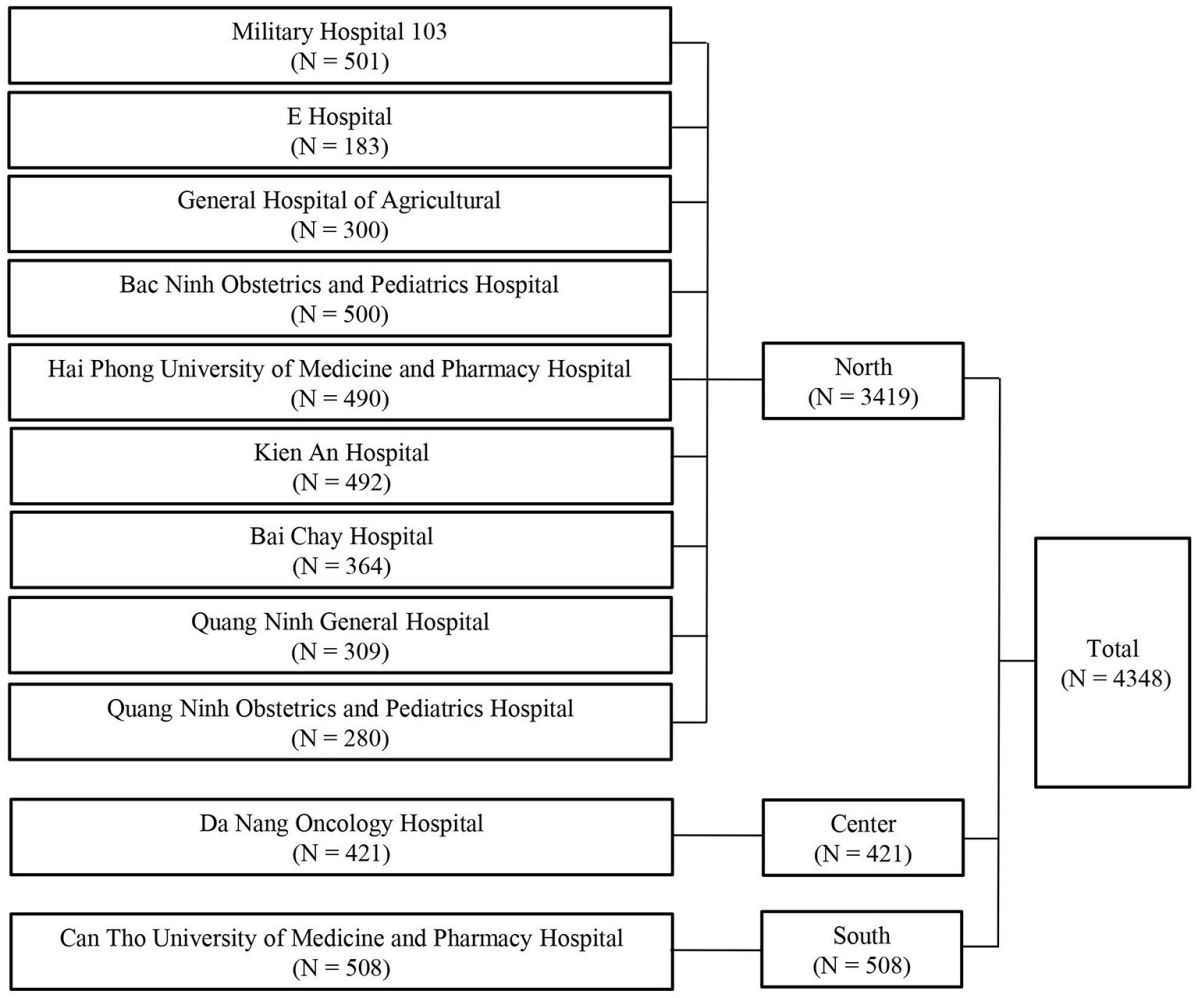
Flow chart of the study sample.

Due to a convenience sample, we aimed to recruit as many participants as possible to reduce the sampling bias and increase the representativeness of the sample. Inclusion criteria were those who visited the outpatient department (OPD) of studied hospitals at the time of this study, aged 18–85, without emergency conditions (e.g., stroke, traumatic brain injury, etc.), and who agreed to participate in the survey. In addition, we excluded patients who had communication difficulties (e.g., deafness or blindness). Finally, we collected and analyzed the data of 4,348 participants. Figure 1 showed the number of patients at each hospital participating in this study (39).

This study was approved by the Ethics Committee of Hanoi University of Public Health (IRB Number: 133/2020/YTCC-HD3).

### Data Collection Procedure

At OPDs of studied hospitals, research assistants (physicians, nurses, and healthcare students) informed patients about the purposes of this study and invited them to participate in the investigation. Informed consent forms were signed before participants carried out the survey. Data was obtained through structured self-administered questionnaires. During the waiting time for examination, participants could take the survey using their smartphone to fill out the online questionnaire via QR code or the printed ones provided at OPDs. The online and printed versions of the questionnaire have the same structure and code. Throughout the time of the survey, research assistants supervised and assisted participants in completing the investigation. Printed questionnaires were checked as participants completed to ensure that all questions were answered. There was no missing data for the online version as all questions included the forced answering option. Therefore, the missing data in this study were minimal. We used the pair-wise deletion method to handle missing data. It took around 10–15 min for each survey. The patient's data was then coded and analyzed for study purposes only.

### Assessments and Measurements

#### Participant's Characteristics

Demographic characteristics were obtained regarding age, gender, marital status (never married vs. ever married), education levels (junior high school or lower vs. senior high school vs. college/university or higher), occupational status (no job vs. has a job), ability to pay for medical care (easy vs. difficult), social status (low vs. middle or high). We calculated body mass index (BMI, kg/m^2^) based on self-reported body weight (kg) and height (cm) and then categorized into three groups: underweight (BMI <18.5), normal weight (18.5 ≤ BMI <25.0), and overweight/obese (BMI ≥ 25.0). The questionnaire used the 14 items of the Charlson Comorbidity Index (51) to assess patients' comorbidities. We then categorized the comorbidity into two groups: “none” vs. “one or more” diseases. Participants with any symptoms resembling COVID-19 (Slike-CV19S), including fever, cough, dyspnea, myalgia, fatigue, sputum production, confusion, headache, sore throat, rhinorrhea, chest pain, hemoptysis, diarrhea, and vomiting (52), were classified as having Slike-CV19S.

#### COVID-19 Induced Lockdown

The national lockdown measure was implemented in Vietnam from April 1–22, 2020 (47, 48). The execution time was categorized into two groups: “under lockdown” vs. “after lockdown,” where patients who conducted the survey in the lockdown period were classified as those under lockdown.

#### Changes in Eating Behavior, Physical Activity, and Mental Health

The questionnaire asked participants about the changes in their current eating and physical activity behaviors compared to those before the COVID-19 pandemic. Patients responded on a five-point scale (never, stopped, less active, unchanged, and more active) for physical activity and a three-point scale (less healthy, unchanged, and healthier) for eating behavior. The World Health Organization (WHO) suggested that individuals should maintain unchanged or improve healthy lifestyles (healthy eating, physical activity) to stay healthy during the pandemic, especially in the lockdown period (46). In this study, participants with “never” and “unchanged” responses to physical activity were those who did not change their physical activity before and during the pandemic. However, a “never” reply was considered a negative behavior. In contrast, an “unchanged” response which means maintaining physical activity at a constant intensity, was considered a positive behavior during the pandemic. Therefore, we categorized physical activity and eating behavior changes into two groups: negative behaviors “never/stopped or less active” vs. positive behaviors “unchanged or more active” for physical activity, and negative behaviors “less healthy” vs. positive behaviors “unchanged or healthier” for eating behavior (43, 53).

We assessed the changes in participants' mental health using the question “How has your mental health changed compared to that before the pandemic?” Patients answered this question with three options, including 1 = “worse,” 2 = “stable,” and 3 = “better.” To ease for analysis, we categorized mental health changes into two groups: “worse” vs. “stable or better” (38).

#### Digital Healthy Diet Literacy and Health Literacy

This study assessed the DDL and health literacy (HL) using the DDL-4 questionnaire and HLS-SF12 questionnaire, respectively. These instruments were developed, validated, and commonly used in previous studies in Vietnam (36, 38, 39, 43, 54–57). In the current study, the Cronbach's α of DDL-4 and HLS-SF12 tools were 0.96 and 0.95, respectively. The patients were asked to rank their difficulty to perform each questionnaire item on four-point responses from 1 = “very difficult” to 4 = “very easy.” We then transformed DDL and HL scores into unified metrics with the ranges from 0 to 50, where participants with a greater DDL score or HL score had better DDL or HL. The formula was documented in prior papers (36, 58).

#### eHealth Literacy

Our study evaluated the eHEALS of participants using an eHealth literacy scale. This instrument consists of eight items, which were validated and utilized in the Vietnamese context (39, 43). The Cronbach's α of eHEALS was 0.96 in this study. Patients ranked their agreement with eight opinions regarding their ability to identify and evaluate health information from online sources. The responses range from 1 = “strongly disagree” to 5 = “strongly agree.” The sum scores were from 8 to 40, in which patients with a greater eHEALS score had better eHEALS.

#### Fear of COVID-19

The fear of COVID-19 scale (FCoV-19S) with seven items was used to assess the fear level of patients. This questionnaire was developed, validated, and used during the pandemic in different countries (59, 60), including Vietnam previous (39, 61). The Cronbach's α of this tool was 0.92 in our study. Patients ranked their consent with seven statements regarding their feelings related to COVID-19 infectability. The possible answers range from 1 = “strongly disagree” to 5 = “strongly agree.” The answers were added up, and the sum scores were from 7 - 35, in which participants with a higher score presented a greater degree of fear of COVID-19.

### Data Analysis

First, we presented independent variables (IVs) as the mean, standard deviation, frequency, and proportion appropriately. Missing data were handled by the pair-wise deletion method. Second, the Chi-squared test and one-way ANOVA test were appropriately performed to compare the proportion of three outcomes (changes in eating behavior, physical activity, and mental health) by different IVs. We used the Benjamini-Hochberg method to decrease the false discovery rate (FDR) when performing multiple comparisons. The raw *p*-values were adjusted to control the level of FDR at 5% using the Benjamini-Hochberg method. Third, we used unadjusted and adjusted logistic regression models to explore the associations of lockdown, DDL, and eHEALS with three outcomes. We chose IVs linked to outcomes at *p* < 0.2 in simple logistic regression models to put in adjusted models. We also performed the Spearman correlation test to check relationships between IVs to avoid multicollinearity. If a moderate or high (*rho* ≥ 0.30) correlation was found between two IVs, we selected a representative one to adjust the final models. Finally, we performed interaction models to examine the modification effects of DDL and eHEALS on the associations between COVID-19 lockdown and three outcomes. If DDL or eHEALS was not associated with outcomes in adjusted logistic regression models, we did not perform the interaction analyses between lockdown and DDL or eHEALS for those outcomes. Unadjusted interaction models were run with three terms that were X1, X2, and X1 × X2. In which X1 is the main effect of lockdown (“Under lockdown × lowest DDL” or “under lockdown × lowest eHEALS”), X2 is the main effects of DDL or eHEALS (“After lockdown × 1-point increment of DDL” or “after lockdown × 1-point increment of eHEALS”), and X1 × X2 is the interaction term (“Under lockdown × 1-point increment of DDL” or “under lockdown × 1-point increment of eHEALS”). Adjusted interaction models were tested with three interaction terms and potential confounders. For visualizing interactions, we conducted the simple slope analyses using PROCESS Marco version 3.5 in SPSS. Before performing simple slope tests, DDL and eHEALS were centralized with a new mean of zero. The graphs were drawn by calculating the expected probability of outcomes by the lockdown variable at three values of DDL or eHEALS (the mean, −1 SD, and +1 SD from the mean). We also reported the coefficients of conditional effects to calculate the odds ratios for the impacts of COVID-19 lockdown on outcomes at three values of DDL or eHEALS. The *p* < 0.05 was defined as a significant level. All analyses were conducted by the IBM SPSS Version 26.0 (IBM Corp, Armonk, NY, United States).

## Results

### Characteristics of Participants

In 4,348 participants, the means of age (year), DDL scores, and eHEALS scores were 42.8 ± 16.7, 25.9 ± 12.2, and 27.9 ± 6.9, respectively. Out of all outpatients, 38.0% (1,654/4,348) were male, 17.8% (772/4,348) were never married, 89.1% (3,874/4,348) had a job, 62.5% (2,712/4,348) found it difficult to pay for medical care, 28.8% (1,254/4,348) had one or more comorbidities, 37.0 % (1,609/4,348) took the survey by online questionnaires. The percentages of outpatients with unchanged or healthier eating behavior, unchanged or more physical activity, and stable or better mental health were 92.5% (4,002/4,348), 42.1% (1,833/4,348), and 62.2% (2,705/4,348), respectively. The proportions of unchanged or healthier eating behavior, unchanged or more physical activity, and stable or better mental health were varied by different categories of age, gender (only for physical activity), marital status, education, occupation, ability to pay for medical care, social status (only for mental health), BMI (only for physical activity), COVID-19 lockdown, Slike-CV19S, comorbidity, health literacy, and fear of COVID-19 (only for mental health) (Benjamini-Hochberg adjusted *p* < 0.05) (Table 1). In addition, two groups “under lockdown” and “after lockdown” had differences in several characteristics, including age, gender, marital status, education, ability to pay for health care, social status, BMI, Slike-CV19S, comorbidity, HL, and fear of COVID-19 (Table 1).

**Table 1:** Characteristics of outpatients by COVID-19 lockdown, and changes in eating behavior, physical activity, mental health (*n* = 4,348).

**Variables**	**Total** **(***n*** = 4,348)**	**COVID-19 lockdown**			**Eating behavior**			**Physical activity**			**Mental health**		
		**After lockdown** **(***n*** = 1,972)**	**Under lockdown** **(***n*** = 2,376)**		**Less healthy** **(***n*** = 325)**	**Unchanged or healthier** **(***n*** = 4,002)**		**Never/stopped or less active** **(***n*** = 2,515)**	**Unchanged or more active** **(***n*** = 1,833)**		**Worse MH** **(***n*** = 1,643)**	**Stable or better MH** **(***n*** = 2,705)**	
	***n*** **(%)**	***n*** **(%)**	***n*** **(%)**	***p*** **^a^**	***n*** **(%)**	***n*** **(%)**	***p*** **^a^**	***n*** **(%)**	***n*** **(%)**	***p*** **^a^**	***n*** **(%)**	***n*** **(%)**	***p*** **^a^**
Age (years), mean (SD)	42.8 (16.7)												
Age groups				<0.001			<0.001			<0.001			<0.001
<60	3,412 (78.5)	1,676 (85.0)	1,736 (73.1)		164 (50.5)	3,233 (80.8)		1,838 (73.1)	1,574 (85.9)		1,068 (65.0)	2,344 (86.7)	
≥60	936 (21.5)	296 (15.0)	640 (26.9)		161 (49.5)	769 (19.2)		677 (26.9)	259 (14.1)		575 (35.0)	361 (13.3)	
Gender				<0.001			0.129			0.009			0.155
Women	2,694 (62.0)	1,312 (66.5)	1,382 (58.2)		188 (57.8)	2,494 (62.3)		1,601 (63.7)	1,093 (59.6)		995 (60.6)	1,699 (62.8)	
Men	1,654 (38.0)	660 (33.5)	994 (41.8)		137 (42.2)	1,508 (37.7)		914 (36.3)	740 (40.4)		648 (39.4)	1,006 (37.2)	
Marital status				<0.001			0.030			<0.001			<0.001
Never married	772 (17.8)	480 (24.4)	292 (12.3)		43 (13.2)	726 (18.2)		343 (13.7)	429 (23.4)		204 (12.4)	568 (21.1)	
Ever married	3,560 (82.2)	1,485 (75.6)	2,075 (87.7)		282 (86.8)	3,261 (81.8)		2,156 (86.3)	1,404 (76.6)		1,438 (87.6)	2,122 (78.9)	
Education level				<0.001			<0.001			<0.001			0.007
Junior high school or	1,007 (23.2)	415 (21.1)	592 (24.9)		99 (30.6)	906 (22.7)		588 (23.4)	419 (22.9)		413 (25.2)	594 (22.0)	
lower													
Senior high school	1,196 (27.5)	465 (23.6)	731 (30.8)		106 (32.7)	1,079 (27.0)		759 (30.2)	437 (23.9)		471 (28.7)	725 (26.9)	
College/university or	2,139 (49.3)	1,088 (55.3)	1,051 (44.3)		119 (36.7)	2,012 (50.3)		1,163 (46.3)	976 (53.3)		758 (46.2)	1,381 (51.1)	
higher													
Occupational status				0.227			0.024			0.008			<0.001
No job	474 (10.9)	202 (10.2)	272 (11.4)		48 (14.8)	423 (10.6)		302 (12.0)	172 (9.4)		245 (14.9)	229 (8.5)	
Having a job	3,874 (89.1)	1,770 (89.8)	2,104 (88.6)		277 (85.2)	3,579 (89.4)		2,213 (88.0)	1,661 (90.6)		1,398 (85.1)	2,476 (91.5)	
Ability to pay for medical care				<0.001			<0.001			<0.001			<0.001
Very or fairly easy	1,626 (37.5)	1,063 (54.0)	1,649 (69.5)		253 (77.8)	2,442 (61.1)		1,698 (67.8)	1,014 (55.3)		1,167 (71.0)	1,545 (57.3)	
Very or fairly difficult	2,712 (62.5)	904 (46.0)	722 (30.5)		72 (22.2)	1,552 (38.9)		808 (32.2)	818 (44.7)		476 (29.0)	1,150 (42.7)	
Social status				0.005			0.129			0.422			<0.001
Low	921 (21.2)	379 (19.3)	542 (22.8)		58 (17.8)	863 (21.6)		543 (21.7)	378 (20.6)		433 (26.4)	488 (18.1)	
Middle or high	3,419 (78.8)	1,589 (80.7)	1,830 (77.2)		267 (82.2)	3,132 (78.4)		1,964 (78.3)	1,455 (79.4)		1,210 (73.6)	2,209 (81.9)	
BMI, kg/m^2^				0.012			0.246			0.003			0.292
Underweight	398 (9.2)	195 (9.9)	203 (8.6)		23 (7.1)	372 (9.3)		205 (8.2)	193 (10.6)		139 (8.5)	259 (9.6)	
Normal weight	3,393 (78.0)	1,496 (76.2)	1,897 (80.0)		253 (78.1)	3,124 (78.3)		1,959 (78.1)	1,434 (78.4)		1,302 (79.5)	2,091 (77.5)	
Overweight/obese	546 (12.6)	274 (13.9)	272 (11.5)		48 (14.8)	496 (12.4)		345 (13.8)	201 (11.0)		197 (12.0)	349 (12.9)	
COVID-19-like symptoms				<0.001			<0.001			<0.001			<0.001
No	2,595 (59.7)	1,343 (68.1)	1,252 (52.7)		115 (35.4)	2,473 (61.8)		1,223 (48.6)	1,372 (74.8)		567 (34.5)	2,028 (75.0)	
Yes	1,753 (40.3)	629 (31.9)	1,124 (47.3)		210 (64.6)	1,529 (38.2)		1,292 (51.4)	461 (25.2)		1,076 (65.5)	677 (25.0)	
Comorbidity				<0.001			<0.001			<0.001			<0.001
None	3,094 (71.2)	1,534 (77.7)	1,560 (65.6)		145 (44.6)	2,938 (73.4)		1,660 (66.0)	1,444 (78.7)		747 (45.4)	2,357 (87.1)	
One or more	1,254 (28.8)	438 (22.3)	816 (34.4)		180 (55.4)	1,064 (26.6)		855 (34.0)	389 (21.3)		896 (54.6)	348 (12.9)	
COVID-19 lockdown		–	–	–			<0.001			<0.001			<0.001
After lockdown	1,972 (45.4)	–	–	–	72 (22.2)	1,899 (47.5)		1,046 (41.6)	926 (50.5)		652 (39.7)	1,320 (48.8)	
Under lockdown	2,376 (54.6)	–	–	–	253 (77.8)	2,103 (52.5)		1,469 (58.4)	907 (49.5)		991 (60.3)	1,385 (51.2)	
Eating behavior changes				<0.001	–	–	–	–	–	–	–	–	–
Less healthy	325 (7.5)	72 (3.7)	253 (10.7)		–	–	–	–	–	–	–	–	–
Unchanged	3,169 (73.2)	1,517 (76.9)	1,652 (69.5)		–	–	–	–	–	–	–	–	–
Healthier	833 (19.3)	382 (19.4)	451 (19.8)		–	–	–	–	–	–	–	–	–
Physical activity changes				<0.001	–	–	–	–	–	–	–	–	–
Never	628 (14.4)	359 (18.2)	269 (11.3)		–	–	–	–	–	–	–	–	–
Stopped	285 (6.6)	101 (5.1)	184 (7.7)		–	–	–	–	–	–	–	–	–
Less active	1,602 (36.8)	586 (29.7)	1,016 (42.8)		–	–	–	–	–	–	–	–	–
Unchanged	1,188 (27.3)	608 (30.8)	580 (24.4)		–	–	–	–	–	–	–	–	–
More active	645 (14.9)	318 (16.2)	327 (13.8)		–	–	–	–	–	–	–	–	–
Mental health changes				<0.001	–	–	–	–	–	–	–	–	–
Worse	1,643 (37.8)	652 (33.1)	991 (41.7)		–	–	–	–	–	–	–	–	–
Stable	2,573 (59.2)	1,255 (63.6)	1,318 (55.5)		–	–	–	–	–	–	–	–	–
Better	132 (3.0)	65 (3.3)	67 (2.8)		–	–	–	–	–	–	–	–	–
HL, mean (SD)	26.5 (10.5)	26.9 (9.9)	26.1 (10.9)	0.026	24.7 (11.1)	26.5 (10.5)	0.004	24.7 (10.5)	28.9 (10.1)	<0.001	22.2 (9.9)	29.0 (9.9)	<0.001
eHEALS, mean (SD)	27.9 (6.9)	27.7 (7.2)	27.9 (6.7)	0.292	27.7 (5.2)	27.8 (7.0)	0.765	26.9 (6.9)	29.1 (6.8)	<0.001	25.3 (6.8)	29.4 (6.5)	<0.001
DDL, mean (SD)	25.9 (12.2)	26.2 (12.1)	25.6 (12.3)	0.105	22.5 (12.6)	26.1 (12.2)	<0.001	23.9 (12.2)	28.7 (11.7)	<0.001	20.8 (11.7)	28.9 (11.5)	<0.001
Fear of COVID-19, mean (SD)	20.6 (5.4)	20.9 (5.6)	20.2 (5.1)	<0.001	20.5 (4.8)	20.6 (5.4)	0.836	20.4 (5.3)	20.7 (5.5)	0.062	21.4 (5.1)	20.0 (5.5)	<0.001

*MH, mental health; SD, standard deviation; DDL, digital healthy diet literacy; eHEALS, eHealth literacy; HL, health literacy*.

a *Results of the Chi-square test or one-way ANOVA test appropriately with Benjamini-Hochberg adjusted p-values*.

### Associations of COVID-19 Lockdown, Digital Healthy Diet Literacy, EHealth Literacy With Changes in Eating Behavior, Physical Activity, and Mental Health

After checking Spearman correlations between IVs, we found that age moderately correlates with education levels (*rho* = −0.34); ability to pay for medical care moderately correlates with social status (*rho* = 0.30); health literacy moderately correlates with comorbidities (*rho* = −0.38), and COVID-19-like symptoms (*rho* = −0.34) (Supplementary Table 1). Therefore, age, gender, ability to pay for medical care, health literacy, and other confounding factors associated with outcomes at *p* <0.2 were added to adjusted logistic regression models (Supplementary Table 2).

The results of adjusted logistic regression models showed that patients under lockdown had a lower likelihood of having unchanged or healthier eating behavior (odds ratio, OR, 0.38; 95% confidence interval, 95% CI, 0.29 to 0.51; *p* < 0.001), unchanged or more physical activity (OR, 0.79; 95% CI, 0.69 to 0.90; *p* < 0.001), and stable or better mental health (OR, 0.77; 95% CI, 0.67 to 0.89; *p* < 0.001) (Table 2). Conversely, participants with a higher DDL had a higher likelihood of having unchanged or healthier eating behavior (OR, 1.02; 95% CI, 1.01 to 1.03; *p* = 0.043), and stable or better mental health (OR, 1.02; 95% CI, 1.01 to 1.03; *p* < 0.001), while participants with a higher eHEALS had a higher likelihood of having unchanged or more physical activity (OR, 1.01; 95% CI, 1.00 to 1.03; *p* = 0.043), and stable or better mental health (OR, 1.06; 95% CI, 1.05 to 1.08; *p* < 0.001) (Table 2).

**Table 2:** Associations of COVID-19 lockdown, digital healthy diet literacy, eHealth literacy with changes in eating behavior, physical activity, and mental health (*n* = 4,348).

**Variables^a^**	**Eating behavior changes^b^**				**Physical activity changes^c^**				**Mental health changes^d^**			
	**Unadjusted model**		**Adjusted model^e^**		**Unadjusted model**		**Adjusted model^f^**		**Unadjusted model**		**Adjusted model^g^**	
	**OR** **(95% CI)**	***p***	**OR** **(95% CI)**	***p***	**OR (95% CI)**	***p***	**OR** **(95% CI)**	***p***	**OR** **(95% CI)**	***p***	**OR** **(95% CI)**	***p***
**COVID-19 lockdown**												
After lockdown	1.00		1.00		1.00		1.00		1.00		1.00	
Under lockdown	0.32 (0.24, 0.41)	<0.001	0.45 (0.33, 0.62)	<0.001	0.69 (0.62, 0.79)	<0.001	0.79 (0.69, 0.90)	<0.001	0.69 (0.61, 0.79)	<0.001	0.77 (0.67, 0.89)	<0.001
Digital healthy diet literacy, 1-score increment	1.02 (1.01, 1.03)	<0.001	1.02 (1.00, 1.04)	0.018	1.03 (1.03, 1.04)	<0.001	1.01 (0.99, 1.02)	0.064	1.06 (1.05, 1.07)	<0.001	1.02 (1.01, 1.03)	<0.001
eHealth literacy, 1-score increment	1.00 (0.98, 1.02)	0.753	0.98 (0.96, 1.01)	0.206	1.05 (1.04, 1.06)	<0.001	1.01 (1.00, 1.03)	0.043	1.10 (1.09, 1.11)	<0.001	1.06 (1.05, 1.08)	<0.001

*OR, odds ratio; CI, confidence interval*.

a *Each independent variable was analyzed separately in different models*.

b *The reference group is “less healthy,” the test group is “unchanged or healthier.”*

c *The reference group is “never/stopped or less active,” the test group is “unchanged or more active.”*

d *The reference group is “worse,” the test group is “stable or better.”*

e *Adjusted for age, gender, marital status, occupational status, ability to pay for medical care, health literacy.*

f *Adjusted for age, gender, marital status, occupational status, ability to pay for medical care, BMI, health literacy, fear of COVID-19.*

g * Adjusted for age, gender, marital status, occupational status, ability to pay for medical care, health literacy, fear of COVID-19*.

### Effect Modification by Digital Healthy Diet Literacy and EHealth Literacy on the Associations of COVID-19 Lockdown With Changes in Eating Behavior, Physical Activity, and Mental Health

**Figure 2:**
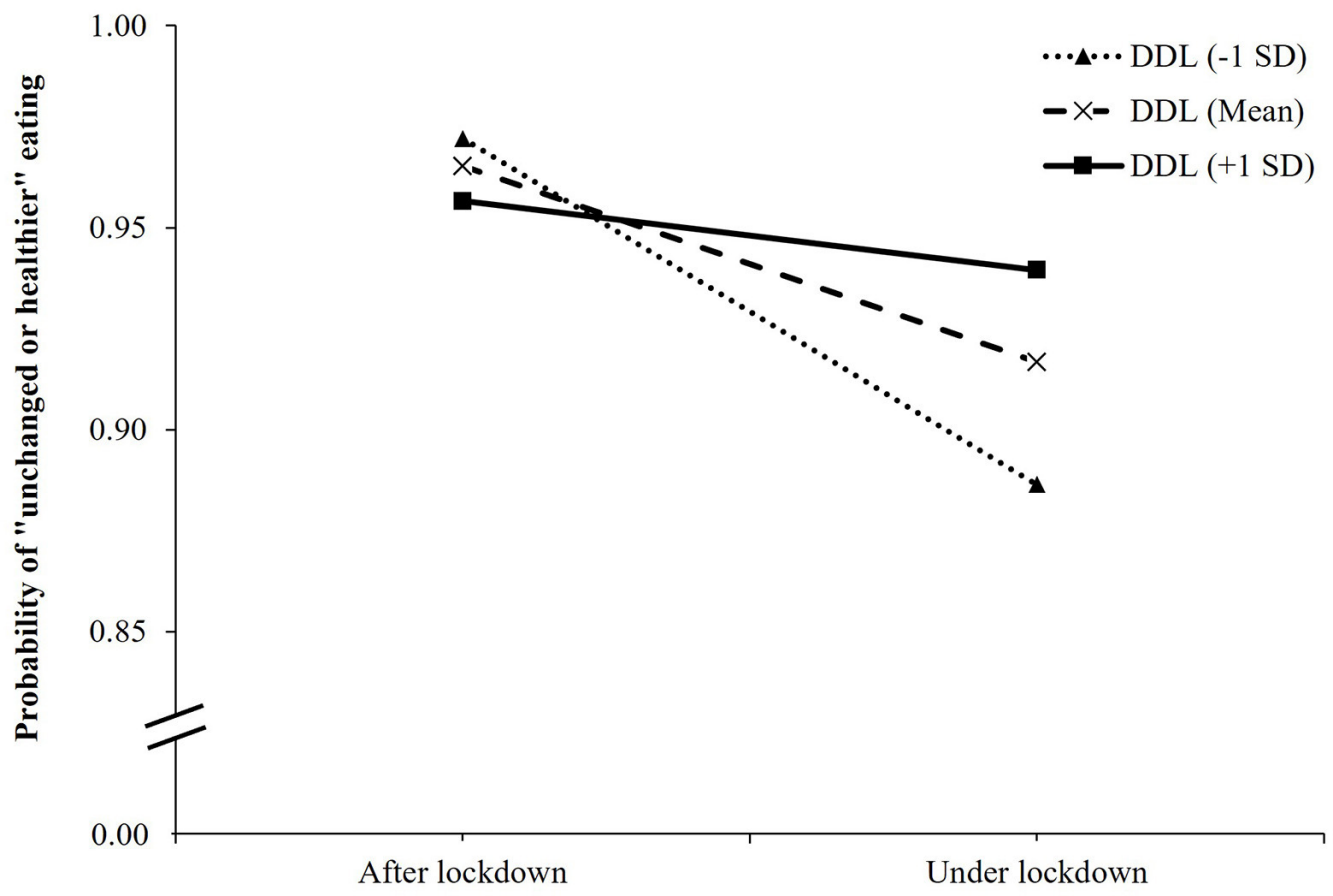
Simple slope plot for interaction between lockdown and digital healthy diet literacy on eating behavior changes among outpatients (*n* = 4,348). DDL, digital healthy diet literacy; SD, standard deviation.

In the interaction model between COVID-19 lockdown and DDL on eating behavior changes, as compared to patients after the lockdown and with the lowest DDL score, those under lockdown and with the lowest DDL score had a lower likelihood of maintaining unchanged or healthier eating behavior (OR, 0.12; 95% CI, 0.06 to 0.23; *p* < 0.001), while those under lockdown and with one DDL-point increment had a higher likelihood of having unchanged or healthier eating behavior (OR, 1.05; 95% CI, 1.02 to 1.07; *p* < 0.001) (Table 3). Figure 2 illustrated the change in the expected probability of unchanged or healthier eating by COVID-19 lockdown at three levels of DDL (the mean, −1 SD, and +1 SD from the mean). The negative impact of COVID-19 lockdown on unchanged or healthier eating was attenuated by higher DDL values from 1 SD below the mean (OR = 0.22, 95% CI, 0.15 to 0.34, *p* < 0.001), the mean (OR = 0.39, 95% CI, 0.30 to 0.54, *p* < 0.001), to 1 SD above the mean (OR = 0.70, 95% CI, 0.50 to 0.99, *p* = 0.048) (Supplementary Table 3). Overall, the significant interaction suggested that when DDL was higher, the inverse association between COVID-19 lockdown and eating behavior changes became weaker.

**Table 3:** Interactions of COVID-19 lockdown with digital healthy diet literacy and eHealth literacy on changes in eating behavior, physical activity, and mental health (*n* = 4,348).

**Variables**	**Eating behavior changes^a^**				**Physical activity changes^b^**				**Mental health changes^c^**			
	**Unadjusted model**		**Adjusted model^d^**		**Unadjusted model**		**Adjusted model^e^**		**Unadjusted model**		**Adjusted model^f^**	
	**OR** **(95% CI)**	***p***	**OR** **(95% CI)**	***p***	**OR** **(95% CI)**	***p***	**OR** **(95% CI)**	***p***	**OR** **(95% CI)**	***p***	**OR** **(95% CI)**	***p***
**Interaction of lockdown with DDL^g^**												
After lockdown × lowest DDL	1.00		1.00		–	–	–	–	1.00		1.00	
Under lockdown × lowest DDL	0.10 (0.05, 0.18)	<0.001	0.13 (0.07, 0.26)	<0.001	–	–	–	–	0.42 (0.31, 0.57)	<0.001	0.44 (0.32, 0.61)	<0.001
After lockdown × DDL, 1-score increment	0.98 (0.97, 1.01)	0.148	0.98 (0.96, 1.01)	0.160	–	–	–	–	1.05 (1.04, 1.06)	<0.001	1.01 (1.00, 1.02)	0.057
Under lockdown × DDL, 1-score increment	1.05 (1.03, 1.07)	<0.001	1.05 (1.03, 1.07)	<0.001	–	–	–	–	1.02 (1.01, 1.03)	<0.001	1.02 (1.01, 1.04)	<0.001
**Interaction of lockdown with eHEALS^h^**												
After lockdown × lowest eHEALS	–	–	–	–	1.00		1.00		1.00		1.00	
Under lockdown × lowest eHEALS	–	–	–	–	0.30 (0.18, 0.51)	<0.001	0.31 (0.18, 0.53)	<0.001	1.55 (0.89, 2.71)	0.122	1.25 (0.69, 2.24)	0.458
After lockdown × eHEALS, 1-score increment	–	–	–	–	1.03 (1.02, 1.05)	<0.001	0.99 (0.98, 1.01)	0.671	1.12 (1.10, 1.14)	<0.001	1.08 (1.06, 1.09)	<0.001
Under lockdown × eHEALS, 1-score increment	–	–	–	–	1.03 (1.01, 1.05)	0.002	1.03 (1.01, 1.05)	0.001	0.97 (0.95, 0.99)	0.002	0.98 (0.96, 1.01)	0.065

*OR, odds ratio; CI, confidence interval; DDL, digital healthy diet literacy; eHEALS, eHealth literacy*.

a *The reference group is “less healthy,” the test group is “unchanged or healthier.”*

b *The reference group is “never/stopped or less active,” the test group is “unchanged or more active.”*

c *The reference group is “worse,” the test group is “stable or better.”*

d *Adjusted for age, gender, marital status, occupational status, ability to pay for medical care, health literacy.*

e *Adjusted for age, gender, marital status, occupational status, ability to pay for medical care, BMI, health literacy, fear of COVID-19.*

f *Adjusted for age, gender, marital status, occupational status, ability to pay for medical care, health literacy, fear of COVID-19.*

g *In Table 2, DDL was not associated with physical activity changes. Thus, the interaction model between lockdown and DDL on physical activity changes was not performed.*

h *In Table 2, eHEALS was not associated with eating behavior changes. Thus, the interaction model between lockdown and eHEALS on eating behavior changes was not performed*.

**Figure 3:**
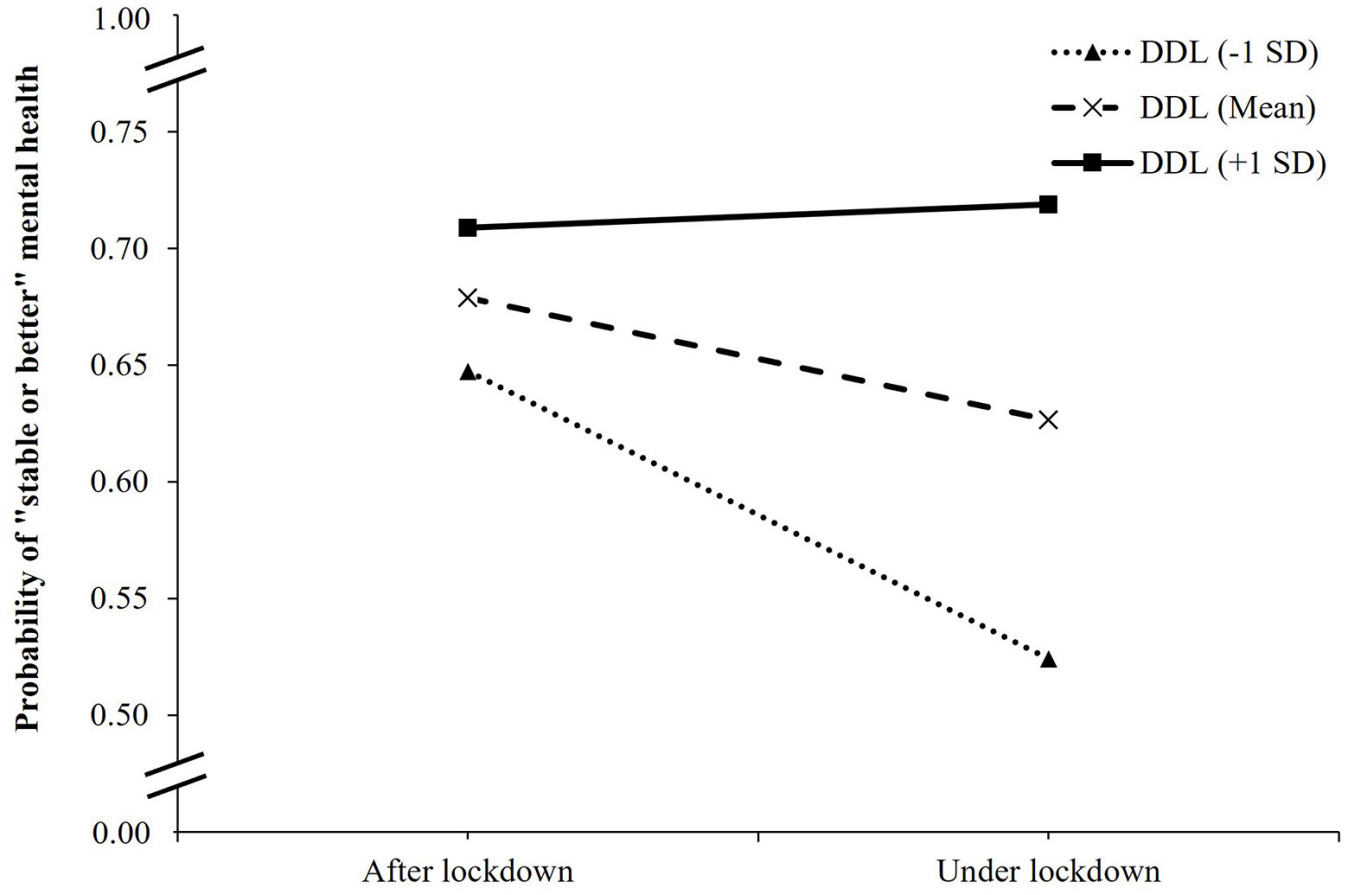
Simple slope plot for interaction between lockdown and digital healthy diet literacy on mental health changes among outpatients (*n* = 4,348). DDL, digital healthy diet literacy; SD, standard deviation.

In the interaction model between COVID-19 lockdown and DDL on mental health changes, as compared patients after the lockdown and with the lowest DDL score, those under lockdown and with the lowest DDL score had lower odds of stable or better mental health (OR, 0.44; 95% CI, 0.32 to 0.61; *p* < 0.001), while those under lockdown and with one DDL-point increment had a higher likelihood of stable or better mental health (OR, 1.02; 95% CI, 1.01 to 1.04; *p* < 0.001) (Table 3). Figure 3 showed the change in the expected probability of stable or better mental health by COVID-19 lockdown at three values of DDL. The negative impact of COVID-19 lockdown on stable or better mental health was attenuated by higher DDL values from 1 SD below the mean (OR = 0.60, 95% CI, 0.50 to 0.72, *p* < 0.001), the mean (OR = 0.79, 95% CI, 0.69 to 0.91, *p* = 0.001), to 1 SD above the mean (OR = 1.05, 95% CI, 0.84 to 1.30, *p* = 0.665) (Supplementary Table 3). Overall, the significant interaction suggested that when DDL was higher, the inverse association between COVID-19 lockdown and mental health changes became weaker.

**Figure 4:**
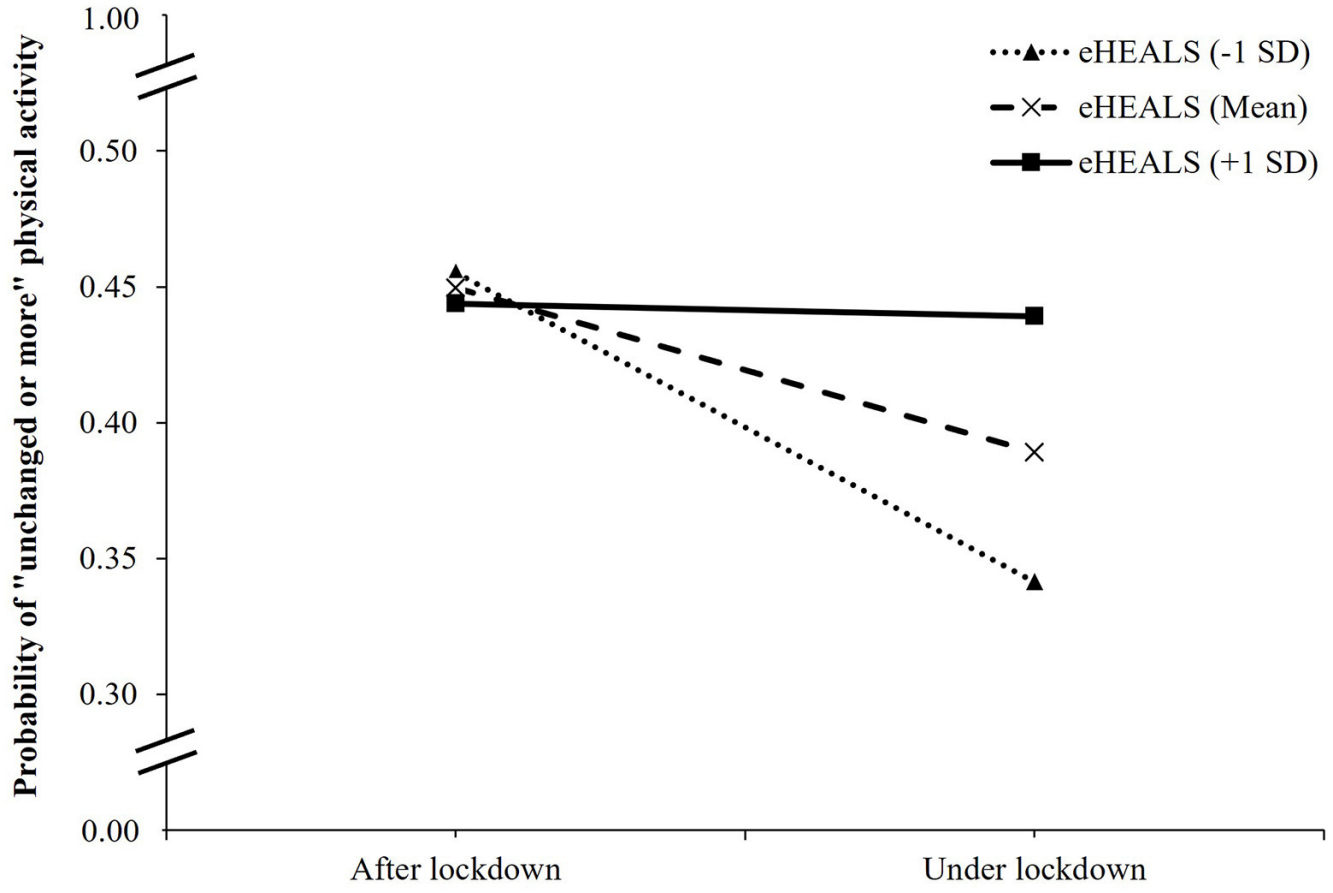
Simple slope plot for interaction between lockdown and eHealth literacy on physical activity changes among outpatients (*n* = 4,348). Note: eHEALS, eHealth literacy; SD, standard deviation.

In the interaction model between COVID-19 lockdown and eHEALS on physical activity changes, as compared to patients after the lockdown and with the lowest eHEALS score, those under lockdown and with the lowest eHEALS score had a lower likelihood of maintaining unchanged or more physical activity (OR, 0.31; 95% CI, 0.18 to 0.53; *p* < 0.001), while those under lockdown and with one eHEALS-point increment had higher odds of having an unchanged or more physical activity (OR, 1.03; 95% CI, 1.01 to 1.05; *p* < 0.001) (Table 3). Figure 4 showed the change in the expected probability of unchanged or more physical activity by COVID-19 lockdown at three values of eHEALS. The negative impact of COVID-19 lockdown on unchanged or more physical activity was attenuated by higher eHEALS values from 1 SD below the mean (OR = 0.62, 95% CI, 0.52 to 0.75, *p* < 0.001), the mean (OR = 0.78, 95% CI, 0.68 to 0.89, *p* < 0.001), to 1 SD above the mean (OR = 0.98, 95% CI, 0.82 to 1.17, *p* = 0.837) (Supplementary Table 3). Overall, the significant interaction suggested that when eHEALS was higher, the inverse association between COVID-19 lockdown and physical activity changes became weaker.

## Discussion

Our findings highlighted the impacts of COVID-19 induced lockdown on changes in eating behavior, physical activity, mental health, and the modification effect of DDL and eHEALS on these associations in outpatients.

The current study found that the lockdown measure was negatively associated with maintaining unchanged or healthier eating behavior, unchanged or more physical activity, and stable or better mental health. A previous systematic review of 64 articles indicated that as compared with pre-lockdown, there was a decrease in physical activity in different populations during the lockdown period (25). Other studies conducted in different countries also showed that the COVID-19 lockdown or home confinement measures had harmful impacts on mental health and health-related behaviors with higher percentages of psychological disorders, physical inactivity, and unhealthy eating habits (11, 19, 23, 24, 62, 63). During the COVID-19 lockdown, restrictions on outside activities and travel, and limited food availability could cause negative changes in physical activity and dietary patterns (20–22, 25). In addition, the lockdown measure could adversely affect patients' mental health due to the difficulty in accessing medical care, delay in treatment, and feelings of boredom or isolation in the home confinement period (10, 11, 35). However, the current study did not study the potential impact of hospitalization and reasons of hospital visits on health-related behaviors, which might have biased the reported results. Therefore, appropriate strategies should be promoted to improve healthy lifestyles and psychological health during the COVID-19 lockdown.

Our study indicated that patients with higher DDL were more likely to have unchanged or healthier eating habits and stable or better psychological health during the pandemic. These findings are consistent with prior studies on front-line medical staff and healthcare students amidst the pandemic (36, 38). Importantly, we found that DDL could help to mitigate the adverse impacts of the lockdown measure on eating behavior and mental health. These findings could be explained that although there was a limited food availability and accessibility, patients with higher DDL may have the ability to evaluate and find for themselves a proper diet and avoid unhealthy foods (e.g., snacks, processed food) through reliable online health information sources during the COVID-19 lockdown. Meanwhile, higher DDL was found to be linked with higher health-related quality of life (39) and healthier eating behavior (36). Furthermore, the beneficial impacts of better dietary intake on psychological health were also documented in previous studies (53, 64, 65). Therefore, by improving the quality of life and diet, DDL could help patients to maintain stable or better mental health during the lockdown period.

Moreover, the results of this study demonstrated that higher eHEALS was associated with a higher likelihood of having unchanged or more physical activity and stable or better mental health. The role of eHEALS in maintaining positive health-related behaviors and protecting mental health has been reported in previous research (40–43). Furthermore, in the interaction model, higher eHEALS could attenuate the harmful effects of COVID-19 induced lockdown on physical activity habits. An explanation for this association is that during the lockdown period, as people have to stay at home and restrict travel, they are more likely to engage in sedentary behaviors and increase screen time (66–68), which in the long term can cause weight gain and chronic diseases (63, 69). Patients with higher eHEALS have higher health awareness, and they have the skills to seek and identify suitable methods to help them maintain physical activity during the home confinement time. Maintaining a healthy diet and staying physically active are essential to improve physical and mental health during the pandemic, especially among people with health problems (46). Therefore, as the lockdown measure was implemented in many countries, enhancing EHL and DDL is critical to help patients to evaluate and identify trusted health information and make the right decisions about their health-related behavior and health.

In the present study, although the magnitude of changes in eating behavior, physical activity, and mental health during and after the lockdown was not substantial, statistically significant results also indicated a negative impact of COVID-19 lockdown on such changes in the short term. In the long run, when the COVID-19 pandemic is uncertain and the lockdown is prolonged, those changes may be more significant and worse without appropriate interventions. Therefore, with a relatively large sample size collected in many hospitals, our research has suggested timely evidence about the adverse impact of lockdowns and protective factors, which may help policymakers develop proper strategies to improve lifestyles and psychological health. In addition, some findings of this study (e.g., associations of DDL and eHEALS with physical activity changes) indicated that a 1-point increase in DDL or eHEALS resulted in a 1% increase in the proportion of unchanged or more physical activity. Although the size of the effects was not large, it would be meaningful if DDL and eHEALS enhancement interventions were implemented comprehensively. It could help people and patients to improve all skills in DDL or eHEALS, not just a specific skill. Therefore, DDL or eHEALS could be enhanced better, leading to a broader and more significant impact of DDL or eHEALS on the outcomes. Besides, mitigating the adverse impact of COVID-19 lockdown may depend on other factors, such as social security policies, food security, unemployment. Therefore, the results of the current study on improving DDL or eHEALS could provide substantive implications, helping patients to improve health-related behaviors and mental health, not only during the lockdown period but also in normal life.

Our study has several drawbacks. First, the causal relationship cannot be inferred from a cross-sectional study. Second, given the urgency of providing timely preliminary evidence for interventions in the initial stage of the pandemic, we used the consecutive convenience sampling method to recruit as many participants as possible. However, we had no data about patients approached in this survey, and only patients who agreed to join and completed the study were recorded. In addition, the total number of patients who visited hospitals during the study period was not recorded and not available on the system. Therefore, we cannot calculate the response rate of this study, which may affect the generalizability of our findings. Third, we used secondary data to analyze the associations in the present study, leading to an increase in the false discovery rate when testing multiple hypotheses on the same sample. Thus, the Benjamini-Hochberg method was used to adjust the *p*-value. Fourth, changes in eating behavior, physical activity, and mental health were assessed using single-item questionnaires, which may be subjective and cause reporting bias. In addition, the current study evaluated the change in physical activity with five answer options: never, stopped, less active, unchanged, and more active. However, the response “never” is an absolute answer that has not changed before and during the pandemic. Besides, it was also assumed that people who were never physically active might improve their behavior over time during the pandemic, while they could not make their physical activity worse than “never.” Due to a cross-sectional study, we classified the “never” response as a negative behavior, which may cause some bias in the classification of physical activity changes. Therefore, results related to physical activity in this study should be applied with caution. Future studies also need to use a better approach for the assessment and classification of these outcomes. Next, we collected data using online and printed versions of the questionnaire, which may affect the results of this study. In addition, because comorbidities and health literacy were moderately correlated (*rho* = −0.38), we chose health literacy to adjust in the final models. However, it is suspected that the findings of this study may be affected by comorbidities. Therefore, we conducted the sensitivity analyses, which added comorbidities (“none” vs. “one or more”) and questionnaire types (“online” vs. “printed”) to adjust in final models. The results showed that the associations and interactions remained significant (Supplementary Tables 4, 5). Finally, other variables influencing outcomes of this study, such as food insecurity, financial difficulty, social support, should be studied in future research.

## Conclusion

The COVID-19 induced lockdown could negatively affect changes in eating behavior, physical activity, and mental health among outpatients. Digital healthy diet literacy and eHealth literacy could help to alleviate the adverse impacts of the COVID-19 induced lockdown on eating behavior, physical activity, and psychological health. Therefore, health organizations and policymakers should promote appropriate interventions to enhance DDL and eHEALS, which help patients to maintain their healthy lifestyles and protect mental health during the lockdown period. However, this study holds several limitations that may undermine the certainty of reported findings.

## Data Availability Statement

The raw data supporting the conclusions of this article will be made available on reasonable request to the corresponding author.

## Ethics Statement

The studies involving human participants were reviewed and approved by Hanoi University of Public Health, Vietnam (IRB Number: 133/2020/YTCC-HD3). The patients/participants provided their written informed consent to participate in this study.

## Author Contributions

ThamN, MN, TP, V-TL, TaN, TL, BD, HD, HN, TH, LP, PN, HoN, TDo, HuN, MT, TL, AT, ThaoN, KN, DP, KP, C-HB, and TDu: conceptualization, methodology, validation, investigation, data curation, and writing review and editing draft. MN, ThamN, and TDu: formal analysis and writing-original draft. MN, TP, and ThaoN: project administration. TDu: supervision and funding acquisition. All authors have read and approved the final manuscript.

## Funding

This research was funded by Hai Phong University of Medicine and Pharmacy and Taipei Medical University (108-6202-008-112; 108-3805-022-400).

## Conflict of Interest

The authors declare that the research was conducted in the absence of any commercial or financial relationships that could be construed as a potential conflict of interest.

## Publisher's Note

All claims expressed in this article are solely those of the authors and do not necessarily represent those of their affiliated organizations, or those of the publisher, the editors and the reviewers. Any product that may be evaluated in this article, or claim that may be made by its manufacturer, is not guaranteed or endorsed by the publisher.
