# Supplementary material for: Negative Impacts of COVID-19 Induced Lockdown on Changes in Eating Behavior, Physical Activity, and Mental Health as Modified by Digital Healthy Diet Literacy and eHealth Literacy
Source: Front Nutr. 2021 Nov 12;8:774328. doi: 10.3389/fnut.2021.774328 (PMC8633895; doi:10.3389/fnut.2021.774328)
Supplement: Supplementary file 1 [file Table_1.DOCX]

**Negative Impacts of COVID-19 Induced Lockdown on Changes in Eating Behavior, Physical Activity, and Mental Health as Modified by Digital Healthy Diet Literacy and eHealth Literacy**

Supplementary Files

[Table S1. Spearman’s correlations (rho) among the studied variables (n= 4348). 2](#_Toc85580571)

[Table S2. Factors associated with changes in eating behavior, physical activity, mental health (n= 4348). 3](#_Toc85580572)

[Table S3. Condition effects and odds ratios of lockdown on three outcomes at values of the moderators (DDL or eHEALS). 4](#_Toc85580573)

[Table S4. Associations of COVID-19 lockdown, digital healthy diet literacy, eHealth literacy with changes in eating behavior, physical activity, and mental health (n= 4348). 5](#_Toc85580574)

[Table S5. Interactions of COVID-19 lockdown with digital healthy diet literacy and eHealth literacy on changes in eating behavior, physical activity, and mental health (n= 4348). 6](#_Toc85580575)

#

# Table S1. Spearman’s correlations (rho) among the studied variables (n= 4348).

| Variables | Age | Gender | Marital status | Education | Occupation | Ability to pay | Social status | Co-  morbidity | BMI | S-COVID-19-S | FCoV-19S | HL |
| --- | --- | --- | --- | --- | --- | --- | --- | --- | --- | --- | --- | --- |
| Gender | .06 |  |  |  |  |  |  |  |  |  |  |  |
| Marital status | .21 | -.03 |  |  |  |  |  |  |  |  |  |  |
| Education | **-.34** | .01 | -.25 |  |  |  |  |  |  |  |  |  |
| Occupation | -.19 | .02 | .06 | .16 |  |  |  |  |  |  |  |  |
| Ability to pay | -.21 | -.03 | -.06 | .26 | .17 |  |  |  |  |  |  |  |
| Social status | -.05 | .00 | .01 | .24 | .18 | **.30** |  |  |  |  |  |  |
| Comorbidity | .29 | .08 | .11 | -.05 | -.07 | -.13 | -.08 |  |  |  |  |  |
| BMI | .01 | .06 | .06 | -.01 | .01 | .04 | .03 | -.01 |  |  |  |  |
| S-COVID-19-S | .22 | .01 | .13 | .05 | -.07 | -.12 | -.01 | **.50** | .01 |  |  |  |
| FCoV-19S | .01 | -.06 | .06 | -.19 | -.03 | .01 | -.06 | -.15 | -.01 | -.21 |  |  |
| HL | -.23 | -.02 | -.18 | .19 | .08 | .19 | .18 | -**.36** | .02 | **-.34** | .070 |  |
| Questionnaire type | -.17 | -.13 | -.05 | .11 | -.03 | .12 | .07 | **-.31** | .01 | -.21 | .19 | .14 |

Abbreviations: BMI, body mass index; S-COVID-19-S, COVID-19-like symptoms; FCoV-19S, fear of COVID-19; HL, health literacy.

# Table S2. Factors associated with changes in eating behavior, physical activity, mental health (n= 4348).

| **Variables** | **Eating behavior changes ^a^** | | **Physical activity changes ^b^** | | **Mental health changes ^c^** | |
| --- | --- | --- | --- | --- | --- | --- |
|  | **OR (95% CI)** | ***p*** | **OR (95% CI)** | ***p*** | **OR (95% CI)** | ***p*** |
| Age groups |  |  |  |  |  |  |
| < 60 | Ref. |  | Ref. |  | Ref. |  |
| ≥ 60 | 0.24 (0.19, 0.30) | <0.001 | 0.45 (0.38, 0.52) | <0.001 | 0.29 (0.25, 0.34) | <0.001 |
| Gender |  |  |  |  |  |  |
| Women | Ref. |  | Ref. |  | Ref. |  |
| Men | 0.83 (0.66, 1.04) | 0.111 | 1.19 (1.05, 1.34) | 0.007 | 0.89 (0.78, 1.01) | 0.068 |
| Marital status |  |  |  |  |  |  |
| Never married | Ref. |  | Ref. |  | Ref. |  |
| Ever married | 0.68 (0.49, 0.95) | 0.025 | 0.52 (0.44, 0.61) | <0.001 | 0.53 (0.44, 0.63) | <0.001 |
| Education level |  |  |  |  |  |  |
| Junior high school or lower | Ref. |  | Ref. |  | Ref. |  |
| Senior high school | 1.12 (0.83, 1.48) | 0.469 | 0.81 (0.68, 0.96) | 0.015 | 1.06 (0.89, 1.26) | 0.489 |
| College/university or higher | 1.85 (1.39, 2.44) | <0.001 | 1.18 (1.01, 1.37) | 0.034 | 1.25 (1.07, 1.45) | 0.005 |
| Occupational status |  |  |  |  |  |  |
| No job | Ref. |  | Ref. |  | Ref. |  |
| Having a job | 1.47 (1.06, 2.02) | 0.020 | 1.32 (1.08, 1.61) | 0.006 | 1.87 (1.54, 2.27) | <0.001 |
| Ability to pay for medical care |  |  |  |  |  |  |
| Very or fairly difficult | Ref. |  | Ref. |  | Ref. |  |
| Very or fairly easy | 2.23 (1.71, 2.92) | <0.001 | 1.69 (1.50, 1.92) | <0.001 | 1.79 (1.57, 2.04) | <0.001 |
| Social status |  |  |  |  |  |  |
| Low | Ref. |  | Ref. |  | Ref. |  |
| Middle or high | 0.79 (0.59, 1.06) | 0.114 | 1.06 (0.92, 1.23) | 0.409 | 1.63 (1.41, 1.89) | <0.001 |
| BMI, kg/m^2^ |  |  |  |  |  |  |
| Underweight (BMI < 18.5) | 1.31 (0.84, 2.03) | 0.229 | 1.29 (1.04, 1.58) | 0.018 | 1.14 (0.91, 1.41) | 0.249 |
| Normal weight (18.5 ≤ BMI < 25.0) | Ref. |  | Ref. |  | Ref. |  |
| Overweight/obese (BMI ≥ 25.0) | 0.84 (0.61, 1.15) | 0.279 | 0.80 (0.66, 0.96) | 0.017 | 1.09 (0.90, 1.32) | 0.364 |
| COVID-19-like symptoms |  |  |  |  |  |  |
| No | Ref. |  | Ref. |  | Ref. |  |
| Yes | 0.34 (0.26, 0.43) | <0.001 | 0.32 (0.28, 0.36) | <0.001 | 0.17 (0.15, 0.20) | <0.001 |
| Comorbidity |  |  |  |  |  |  |
| None | Ref. |  | Ref. |  | Ref. |  |
| One or more | 0.29 (0.23, 0.37) | <0.001 | 0.52 (0.45, 0.60) | <0.001 | 0.12 (0.11, 0.14) | <0.001 |
| Questionnaire types |  |  |  |  |  |  |
| Printed | Ref. |  | Ref. |  | Ref. |  |
| Online | 2.91 (2.18, 3.89) | <0.001 | 1.14 (1.01, 1.29) | 0.038 | 1.52 (1.34, 1.73) | <0.001 |
| Health literacy, 1-score increment | 1.02 (1.01, 1.03) | 0.003 | 1.04 (1.03, 1.05) | <0.001 | 1.07 (1.06, 1.08) | <0.001 |
| Fear of COVID-19, 1-score increment | 1.00 (0.98, 1.02) | 0.836 | 1.01 (0.99, 1.02) | 0.051 | 0.95 (0.94, 0.96) | <0.001 |

^a^ The reference group is ‘less healthy’, the test group is ‘unchanged or healthier’.

^b^ The reference group is ‘never/stopped or less active’, the test group is ‘unchanged or more active’

^c^ The reference group is ‘worse’, the test group is ‘stable or better’.

# Table S3. Condition effects and odds ratios of lockdown on three outcomes at values of the moderators (DDL or eHEALS).

| **Lockdown ^a^** | **Healthier eating behavior** | | | **Unchanged or more physical activity** | | | **Stable or better mental health** | | |
| --- | --- | --- | --- | --- | --- | --- | --- | --- | --- |
|  | **b (95% CI)** | **OR (95% CI)** | ***p ^b^*** | **b (95% CI)** | **OR (95% CI)** | ***p ^c^*** | **b (95% CI)** | **OR (95% CI)** | ***p ^d^*** |
| **Lockdown at values of DDL ^e^** |  |  |  |  |  |  |  |  |  |
| - 1 SD (DDL = -12.25) | -1.49  (-1.89, -1.09) | 0.22  (0.15, 0.34) | <0.001 | - | - | - | -0.51  (-0.70, -0.32) | 0.60  (0.50, 0.72) | <0.001 |
| Mean (DDL = 0) | -0.92  (-1.21, -0.63) | 0.39  (0.30, 0.54) | <0.001 | - | - | - | -0.23  (-0.37, -0.09) | 0.79  (0.69, 0.91) | 0.001 |
| + 1 SD (DDL = 12.25) | -0.35  (-0.69, -0.01) | 0.70  (0.50, 0.99) | 0.048 | - | - | - | 0.05  (-0.17, 0.27) | 1.05  (0.84, 1.30) | 0.665 |
| **Lockdown at values of eHEALS ^e^** |  |  |  |  |  |  |  |  |  |
| - 1 SD (eHEALS = -6.92) | - | - | - | -0.48  (-0.66, -0.29) | 0.62  (0.52, 0.75) | <0.001 | - | - | - |
| Mean (eHEALS = 0) | - | - | - | -0.25  (-0.38, -0.12) | 0.78  (0.68, 0.89) | <0.001 | - | - | - |
| + 1 SD (eHEALS = 6.92) | - | - | - | -0.02  (-0.20, 0.16) | 0.98  (0.82, 1.17) | 0.837 | - | - | - |

Abbreviations: b, coefficient of condition effects; OR, odds ratio; CI, confidence interval; SD, standard deviation; DDL, digital healthy diet literacy; eHEALS, eHealth literacy.

^a^ The reference group is ‘after lockdown’, the test group is ‘under lockdown’.

^b^ Adjusted for age, gender, marital status, occupational status, ability to pay for medical care, health literacy.

^c^ Adjusted for age, gender, marital status, occupational status, ability to pay for medical care, BMI, health literacy, fear of COVID-19.

^d^ Adjusted for age, gender, marital status, occupational status, ability to pay for medical care, health literacy, fear of COVID-19.

^e^ DDL and eHEALS were centered by subtracting the mean from each DDL or eHEALS value.

# Table S4. Associations of COVID-19 lockdown, digital healthy diet literacy, eHealth literacy with changes in eating behavior, physical activity, and mental health (n= 4348).

| Variables ^a^ | Eating behavior changes ^b^ | | | | Physical activity changes ^c^ | | | | Mental health changes ^d^ | | | |
| --- | --- | --- | --- | --- | --- | --- | --- | --- | --- | --- | --- | --- |
|  | Unadjusted model | | Adjusted model ^e^ | | Unadjusted model | | Adjusted model ^f^ | | Unadjusted model | | Adjusted model ^g^ | |
|  | OR (95% CI) | *p* | OR (95% CI) | *p* | OR (95% CI) | *p* | OR (95% CI) | *p* | OR (95% CI) | *p* | OR (95% CI) | *p* |
| COVID-19 induced lockdown |  |  |  |  |  |  |  |  |  |  |  |  |
| After lockdown | Ref. |  | Ref. |  | Ref. |  | Ref. |  | Ref. |  | Ref. |  |
| Under lockdown | 0.32  (0.24, 0.41) | <0.001 | 0.47  (0.34, 0.64) | <0.001 | 0.69  (0.62, 0.79) | <0.001 | 0.72  (0.63, 0.83) | <0.001 | 0.69  (0.61, 0.79) | <0.001 | 0.84  (0.71, 0.98) | 0.030 |
| DDL, 1-score increment | 1.02  (1.01, 1.03) | <0.001 | 1.02  (1.01, 1.03) | 0.036 | 1.03  (1.03, 1.04) | <0.001 | 1.01  (0.99, 1.02) | 0.067 | 1.06  (1.05, 1.07) | <0.001 | 1.02  (1.01, 1.03) | <0.001 |
| eHEALS, 1-score increment | 1.00  (0.98, 1.02) | 0.753 | 0.98  (0.96, 1.00) | 0.078 | 1.05  (1.04, 1.06) | <0.001 | 1.02  (1.00, 1.03) | 0.014 | 1.10  (1.09, 1.11) | <0.001 | 1.04  (1.03, 1.06) | <0.001 |

Abbreviations: OR, odds ratio; CI, confidence interval; DDL, digital healthy diet literacy; eHEALS, eHealth literacy.

^a^ Each independent variable was analyzed separately in different models.

^b^ The reference group is ‘less healthy’, the test group is ‘unchanged or healthier’.

^c^ The reference group is ‘never/stopped or less active’, the test group is ‘unchanged or more active’.

^d^ The reference group is ‘worse’, the test group is ‘stable or better’.

^e^ Adjusted for age, gender, marital status, occupational status, ability to pay for medical care, comorbidity, questionnaire types, health literacy.

^f^ Adjusted for age, gender, marital status, occupational status, ability to pay for medical care, BMI, comorbidity, questionnaire types, health literacy, fear of COVID-19.

^g^ Adjusted for age, gender, marital status, occupational status, ability to pay for medical care, comorbidity, questionnaire types, health literacy, fear of COVID-19.

# Table S5. Interactions of COVID-19 lockdown with digital healthy diet literacy and eHealth literacy on changes in eating behavior, physical activity, and mental health (n= 4348).

| Variables | Eating behavior changes ^a^ | | | | Physical activity changes ^b^ | | | | Mental health changes ^c^ | | | |
| --- | --- | --- | --- | --- | --- | --- | --- | --- | --- | --- | --- | --- |
|  | Unadjusted model | | Adjusted model ^d^ | | Unadjusted model | | Adjusted model ^e^ | | Unadjusted model | | Adjusted model ^f^ | |
|  | OR (95% CI) | *p* | OR (95% CI) | *p* | OR (95% CI) | *p* | OR (95% CI) | *p* | OR (95% CI) | *p* | OR (95% CI) | *p* |
| *Interaction of lockdown with DDL* | | | | | | | | | | | | |
| After lockdown × lowest DDL | Ref. |  | Ref. |  | - | - | - | - | Ref. |  | Ref. |  |
| Under lockdown × lowest DDL | 0.10  (0.05, 0.18) | <0.001 | 0.15  (0.08, 0.29) | <0.001 | - | - | - | - | 0.42  (0.31, 0.57) | <0.001 | 0.61  (0.43, 0.86) | 0.005 |
| After lockdown × DDL, 1-score increment | 0.98  (0.97, 1.01) | 0.148 | 0.98  (0.96, 1.01) | 0.173 | - | - | - | - | 1.05  (1.04, 1.06) | <0.001 | 1.02  (1.00, 1.03) | 0.014 |
| Under lockdown × DDL, 1-score increment | 1.05  (1.03, 1.07) | <0.001 | 1.05  (1.02, 1.07) | <0.001 | - | - | - | - | 1.02  (1.01, 1.03) | <0.001 | 1.01  (1.00, 1.02) | 0.044 |
| *Interaction of lockdown with eHEALS* | | | | | | | | | | | | |
| After lockdown × lowest eHEALS | - | - | - | - | Ref. |  | Ref. |  | Ref. |  | Ref. |  |
| Under lockdown × lowest eHEALS | - | - | - | - | 0.30  (0.18, 0.51) | <0.001 | 0.25  (0.15, 0.44) | <0.001 | 1.55 (0.89, 2.71) | 0.122 | 1.79  (0.98, 3.27) | 0.057 |
| After lockdown × eHEALS, 1-score increment | - | - | - | - | 1.03  (1.02, 1.05) | <0.001 | 0.99  (0.98, 1.01) | 0.815 | 1.12 (1.10, 1.14) | <0.001 | 1.06  (1.04, 1.08) | <0.001 |
| Under lockdown × eHEALS, 1-score increment | - | - | - | - | 1.03  (1.01, 1.05) | 0.002 | 1.04  (1.02, 1.06) | <0.001 | 0.97 (0.95, 0.99) | 0.002 | 0.97  (0.95, 1.00) | 0.056 |

Abbreviations: OR, odds ratio; CI, confidence interval; DDL, digital healthy diet literacy; eHEALS, eHealth literacy.

^a^ The reference group is ‘less healthy’, the test group is ‘unchanged or healthier’.

^b^ The reference group is ‘never/stopped or less active’, the test group is ‘unchanged or more active’.

^c^ The reference group is ‘worse’, the test group is ‘stable or better’.

^d^ Adjusted for age, gender, marital status, occupational status, ability to pay for medical care, comorbidity, questionnaire types, health literacy.

^e^ Adjusted for age, gender, marital status, occupational status, ability to pay for medical care, BMI, comorbidity, questionnaire types, health literacy, fear of COVID-19.

^f^ Adjusted for age, gender, marital status, occupational status, ability to pay for medical care, comorbidity, questionnaire types, health literacy, fear of COVID-19.

^g^ In Table S3, DDL was not associated with physical activity changes. Therefore, the interaction between lockdown and DDL on physical activity changes was not performed.

^h^ In Table S3, eHEALS was not associated with eating behavior changes. Therefore, the interaction between lockdown and eHEALS on eating behavior changes was not performed.
